# Diversity of intertidal, epibiotic, and fouling barnacles (Cirripedia, Thoracica) from Gujarat, northwest India

**DOI:** 10.3897/zookeys.1026.60733

**Published:** 2021-03-25

**Authors:** Jigneshkumar N. Trivedi, Mahima Doshi, Krupal J. Patel, Benny K.K. Chan

**Affiliations:** 1 Department of Life Sciences, Hemchandracharya North Gujarat University, Patan-384265, Gujarat, India Hemchandracharya North Gujarat University Gujarat India; 2 Marine Biodiversity and Ecology Laboratory, Department of Zoology, The Maharaja Sayajirao University of Baroda, Vadodara-390002, Gujarat, India The Maharaja Sayajirao University of Baroda Gujarat India; 3 Biodiversity Research Center, Academia Sinica, Taipei 115, Taiwan Biodiversity Research Center, Academia Sinica Taipei Taiwan

**Keywords:** Arabian Sea, biogeography, ecoregions, new records, provinces

## Abstract

The present work studied the diversity of intertidal, epibiotic, and fouling barnacles in the state of Gujarat, northwest India. In total, eleven species belonging to eight genera and five families were recorded in the present study. The Arabian intertidal species *Tetraclita ehsani* Shahdadi, Chan & Sari, 2011 and *Chthamalus barnesi* Achituv & Safriel, 1980 are common in the high- and mid-intertidal rocky shores of Gujarat suggesting that the Gujarat barnacle assemblages are similar to the assemblages in the Gulf of Oman Ecoregion. The biogeographical boundary between the Gulf of Oman and Western Indian ecoregions for barnacles should probably extend southward towards the waters adjacent to Mumbai, where Indo-Pacific species of intertidal barnacles dominate. This study provides the first reports of the common widely distributed balanomorph barnacles *Striatobalanus tenuis* (Hoek, 1883), *Tetraclitella karandei* Ross, 1971, *Amphibalanus reticulatus* (Utinomi, 1967), and lepadid barnacle *Lepas anatifera* Linnaeus, 1758 in Gujarat, as well as of the chthamalid barnacle *Chthamalus barnesi* in India.

## Introduction

Barnacles are marine crustaceans that inhabit a diverse range of substrates, including rocks, molluscan shells, corals, sponges, mangrove roots and leaves, turtle shells, and whale skin (Chan and Høeg 2015; Kim et al. 2020). Fossilized barnacle shells are often used to study the past environment (Bianucci et al. 2006a, b; Collareta et al. 2016a, b, 2018; Buckeridge et al. 2018, 2019). Burmeister (1834) was the first to classify barnacles into cirripedes, which later attracted the attention of numerous taxonomists including Charles Darwin (Anderson 1994). Barnacles have ecological and economic importance, as some species are biofoulers and others are considered seafood in some countries (Walker 1972; Newman and Abbott 1980; Santhakumaran and Sawant 1991; Rawangkul et al. 1995; Molnar et al. 2008; Sophia Rani et al. 2010; Holm 2012). More than 1400 species of barnacles are listed globally (Chan et al. 2009), and most are abundant along the intertidal and subtidal zones of temperate and tropical regions (Frith et al. 1976; Brickner and Høeg 2010; Brickner et al. 2010; Sophia Rani et al. 2010; Chen et al. 2012, 2014; Hayashi 2013; Yu et al. 2016). Taxonomic study of the Indian barnacle fauna dates back to the systemic work carried out by Darwin (1854), which was followed by several important studies in the 1900s (Annandale 1906, 1909, 1914; Nilsson-Cantell 1938; Daniel 1956, 1972, 1981). Fernando (2006) prepared a monograph on the barnacles of India in which he recorded 70 species of barnacles from Indian waters.

**Figure 1.**
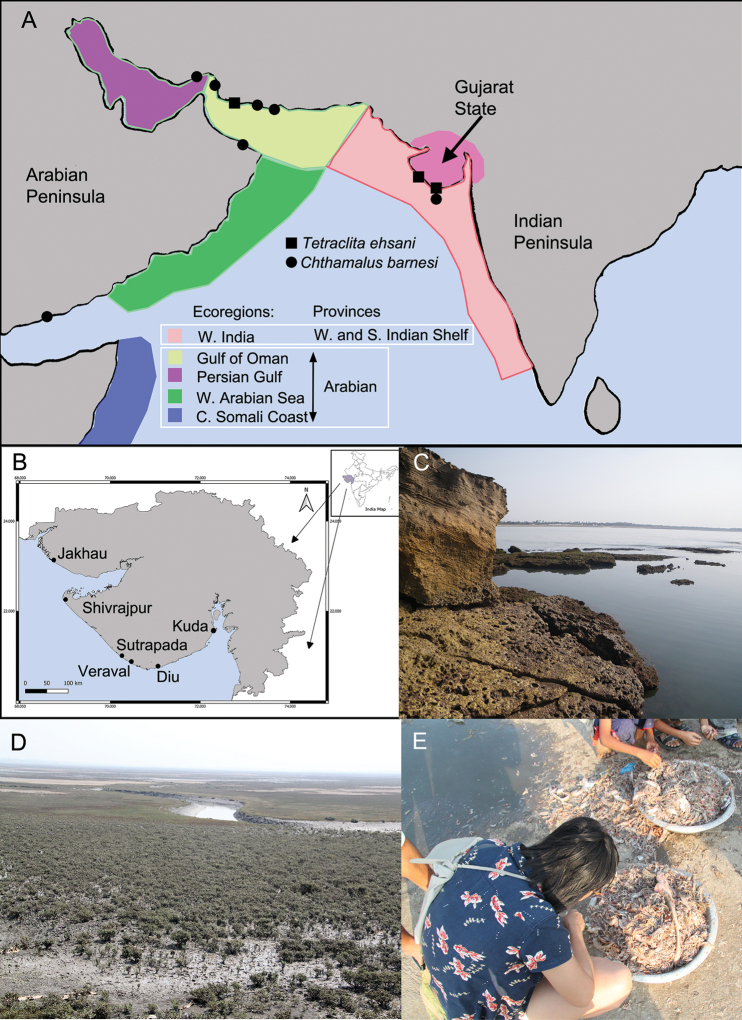
**A** map of the Arabian Sea and Indian Ocean showing the definition of ecoregions and provinces according to Spalding et al. (2007). The distribution records of *Chthamalus barnesi* and *Tetraclita ehsani* in the Persian Gulf, Gulf of Oman (Shahdadi and Sari 2011, Shahdadi et al. 2011), and in Gujarat (present study) are also plotted **B** map of Gujarat showing the sampling locations of barnacles collected in the present study **C** sandstone rocky intertidal at Diu, Gujarat **D** mangroves are common habitats in Gujarat and with *Amphibalanus amphitrite* on rocks **E** traditional Indian fish markets, where decapods with barnacles can be collected from the bulk by-catches gathered by fishermen.

Spalding et al. (2007) classified the world’s biogeographical provinces and ecoregions within provinces. The Persian Gulf, Gulf of Oman, and Arabian Sea belong to two provinces (Fig. 1A): the Arabian Province includes the Persian Gulf, Gulf of Oman, Western Arabian Sea, and Central Somali Coast Ecoregions. The West and South India Shelf Province covers the western and southern coastlines of India and Sri Lanka and is divided into the Western Indian Ecoregion and South India and Sri Lanka Ecoregion. Gujarat is the westernmost state of India and contains 1650 km of coastline (Fig. 1A, B). It possesses a variety of coastal habitats, including mangroves, coral reefs, rocky shores, mudflats, sandy shores, and estuaries (Fig. 1C–E; Trivedi et al. 2015). In the present work, we describe the species recorded in Gujarat and discussed the similarity in the assemblages of Gujarat between the Gulf of Oman and Western Indian Ecoregions.

## Materials and methods

### Study area

The coastal area of Gujarat is mainly divided into three major coastline regions: Saurashtra Coast, Gulf of Khambhat, and Gulf of Kachchh (Trivedi et al. 2015; Fig. 1B). Barnacle specimens were collected from five different sites: Jakhau (23°11.30'N, 68°37.35'E), Sutrapada (20°50.38'N, 70°28.46'E), Veraval (20°54.60'N, 70°21.13'E), Diu (20°42.88'N, 70°53.17'E) and Kuda Beach, Bhavnagar (21°37.70'N, 72°18.40'E) (Fig. 1B).

### Specimen collection and identification

Specimens were collected during low tides using a hammer and chisel from 2010–2020. Photographs of live specimens were taken in the field and then preserved in 10% formalin or 95% ethanol for further analysis in the laboratory. In the laboratory, barnacles were first identified based on their shell morphometry using a stereomicroscope. Specimens were gently dissected from their shell under a stereomicroscope with camera for specimen identification. The following barnacle parts were dissected: mouthparts (maxilla, maxillule, mandible, mandibulatory palp, and labrum), tergum, and scutum. The identification key of Chan et al. (2009) was used for basic taxonomic identification as well as for general terminologies of shell morphology and other important characters. All the specimens were deposited into the Zoological Reference Collection (**LFSc.ZRC**), Department of Life Sciences, Hemchandracharya North Gujarat University, Patan, Gujarat, India and Biodiversity Research Museum (**ASIZCR**), Academia Sinica, Taiwan. Rostral-carinal basal diameter of shells (**BD**) of sessile barnacles and capitulum length (**CL**, from the basal margin of scutum to apex of tergum) of stalked barnacles were measured to the nearest 0.01 mm.

### Zonation pattern of rocky intertidal species at Diu, Gujarat

To examine the zonation of intertidal barnacles, stratified transect surveys were conducted in two rocky shores of Nagoa Beach in Diu (20°42.12'N, 70°55.0217'E and 20°42.17'N, 70° 53.94'E). The maximum tidal range at Diu is approximately 2.5 metres. At each shore, 10-m-long stretches of shoreline were selected. Sampling was conducted at the highest tidal level at which chthamalid barnacles were found (2 m above Chart Datum, C.D.). Subsequent tidal levels were sampled at 0.5 m vertical intervals, 1.5 m above C.D. and 1.0 m above C.D. At each tidal level, ten random 0.25 × 0.25 m quadrats were established and the number of individuals of each species of barnacles was scored.

## Results

A total of eleven barnacle species was recorded, belonging to six genera and five families. The common species recorded belonged to the family Balanidae (3 species, 2 genera), followed by Lepadidae (2 species, 1 genus), Chthamalidae (2 species, 2 genus), Tetraclitidae (2 species, 2 genus), Archaeobalanidae (1 species), and Chelonibiidae (1 species). *Chthamalus barnesi* Achituv & Safriel, 1980 was reported for the first time from India. *Lepas anatifera* Linnaeus, 1758 was reported for the first time from the west coast of India, while *Tetraclitella karandei* Ross, 1971, *Striatobalanus tenuis* (Hoek, 1883) and *Amphibalanus reticulatus* (Utinomi, 1967) were reported for the first time from the state of Gujarat.

### Systematics

#### Cirripedia Burmeister, 1834

**Thoracica Darwin, 1854**

**Sessilia Lamarck, 1818**

**Balanomorpha Pilsbry, 1916**

**Balanoidea Leach, 1817**

**Archaeobalanidae Newman & Ross, 1976**

##### *Striatobalanus* Hoek, 1913

###### Striatobalanus tenuis

Taxon classificationAnimaliaSessiliaArchaeobalanidae

(Hoek, 1883)

62AC482D-2C57-5C62-80C2-878DB17D65CC

Figures 2A–C , 4

####### Examined material.

four specimens (BD: 9.43–13.59 mm), LFSc.ZRC-157 (2 specimens on *Murex ternispina* Lamarck, 1822, one specimen on *Babylonia spirata* Linnaeus, 1758, and one specimen on *Bufonaria echinata* Link, 1807), Jakhau, Kachchh (23°11.30'N, 68°37.35'E), 9 January 2020, Gujarat, India, sandy shore, leg. M. Doshi.

####### Diagnosis

**Figure 2.**
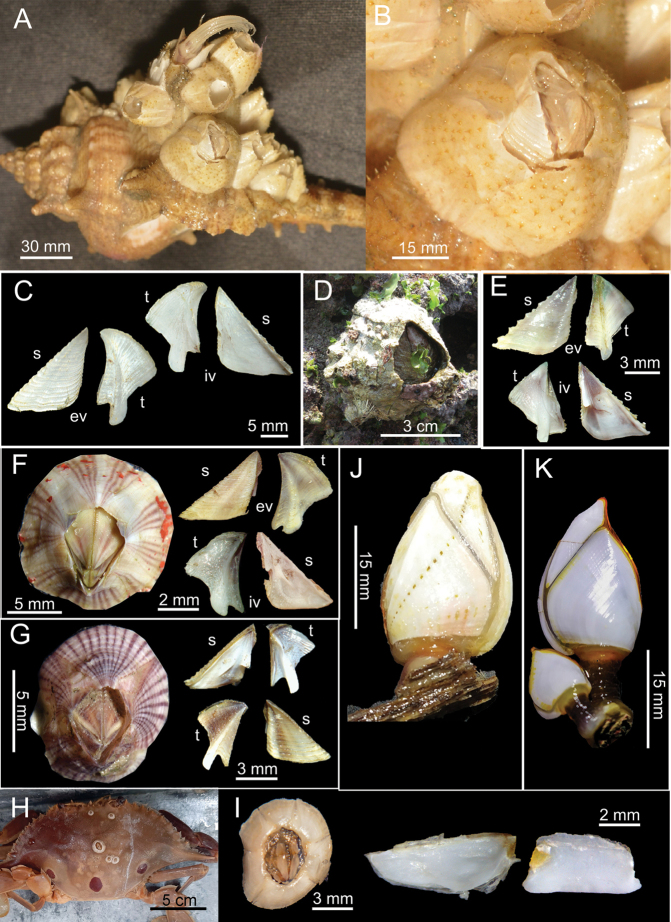
Gujarat barnacles **A***Striatobalanus tenuis* (Hoek, 1883) on gastropod shell (*Murex ternispina* Lamarck, 1822) **B** top view of *Striatobalanus tenuis* (BD: 11.28 mm) LFSc.ZRC-157 **C** external and internal view of scutum and tergum **D***Megabalanus tintinnabulum* on shores LFSc.ZRC-182 **E** external and internal view of scutum and tergum **F***Amphibalanus amphitrite* (Darwin, 1854), top view, (BD: 12.38 mm) LFSc.ZRC-181, external and internal views of scutum and tergum **G***Amphibalanus reticulatus* (Utinomi, 1967), top view, (BD: 14.99 mm) LFSc.ZRC-158, external and internal views of scutum and tergum **H***Chelonibia testudinaria* (Linnaeus, 1758) on crab *Portunus sanguinolentus***I***Chelonibia testudinaria* (Linnaeus, 1758), top view (BD: 5.33 mm) LFSc.ZRC-159, internal view of scutum and tergum **J***Lepas anatifera* Linnaeus, 1758, (CL: 16.39 mm) LFSc.ZRC-162, a. Side view of capitulum **K***Lepas anserifera* Linnaeus, 1758, (CL: 16.28 mm) LFSc.ZRC-183, Side view of capitulum.

**(modified from Chan et al. 2009).** Shell composed of six plates, conical, white, orifice deeply toothed (Fig. 2B). Scutum triangular, strongly sculptured on dorsal surface (Fig. 2C). Tergum triangular with long and narrow spur, scutal margin concave, medial furrow present on dorsal side of tergum (Fig. 2C). Scutal and tergal outer surfaces striated longitudinally. Maxilla triangular, covered with dense setae (Fig. 4A). Maxillule not notched, with two large setae on upper region (Fig. 4B). Mandible with five teeth excluding inferior angle, inferior angle blunt, 2^nd^ and 3^rd^ teeth bi-dentate (Fig. 4C–E). Mandibulatory palp rounded with setae at tip and superior margin (Fig. 4F). Labrum bullate shaped with distinct and deep notch having two prominent teeth on each side of cutting edge (Fig. 4G, H).

**Figure 3.**
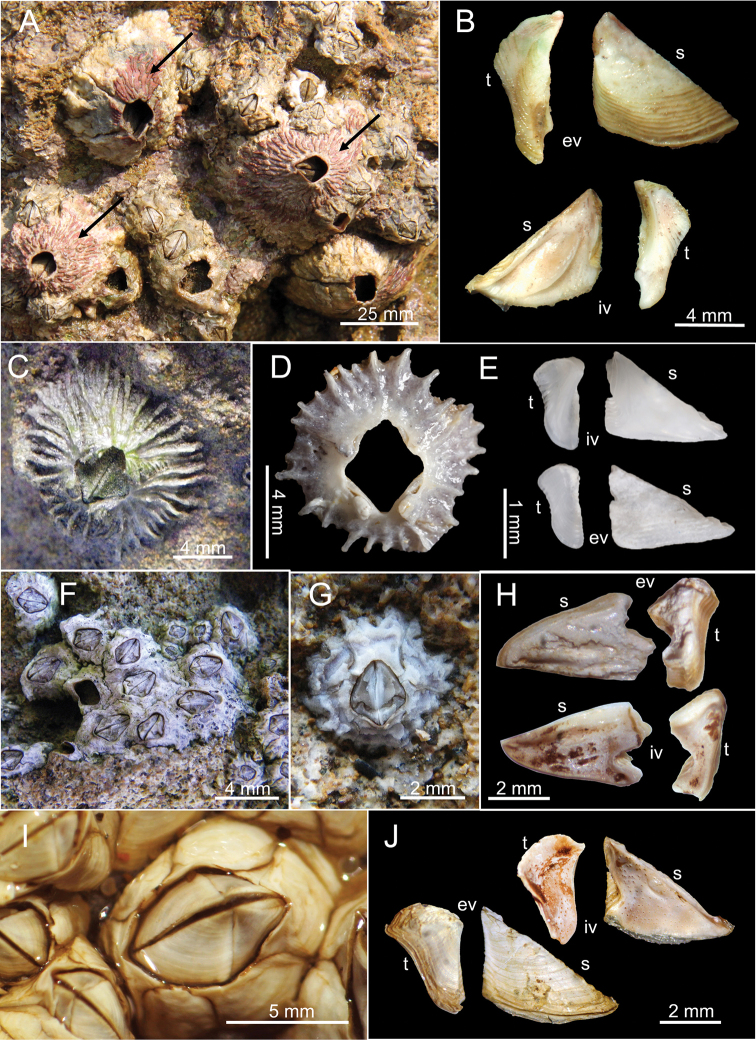
**A***Tetraclita ehsani*LFSc.ZRC-184 on shores of Diu **B***Tetraclita ehsani*, external and internal view of scutum and tergum **C***Tetraclitella karandei* ASIZCR000454 on shores at Diu **D** shell of *T. karandei***E***Tetraclitella karandei* external and internal view of scutum and tergum **F***Chthamalus barnesi* on shores **G** close up view of *C. barnesi*LFSc.ZRC-160 **H** Internal and external view of scutum and tergum of *C. barnesi***I***Microeuraphia withersi*LFSc.ZRC-161 (BD: 6.01 mm) **J** internal and external view of scutum and tergum of *M. withersi*.

**Figure 4.**
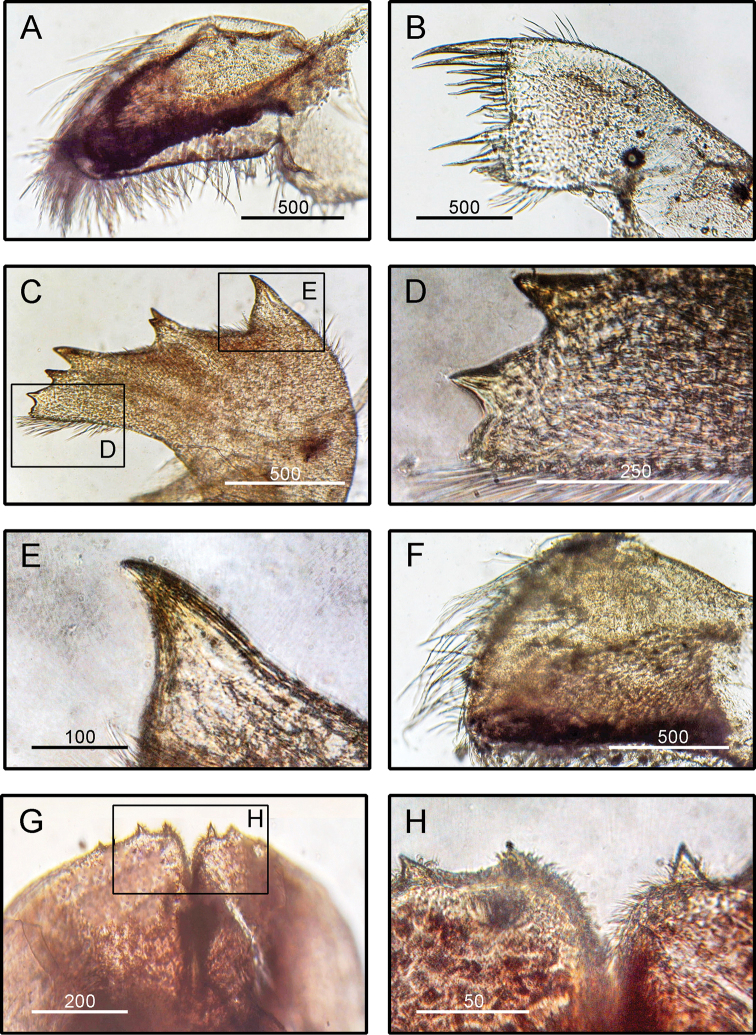
*Striatobalanus tenuis* (Hoek, 1883), (BD: 11.28 mm) LFSc.ZRC-157, Light microscopy on mouth parts **A** maxilla **B** maxillule **C** mandible **D** close up on the inferior angle of mandible **E** close up on the teeth of mandible **F** mandibulatory palp **G** labrum **H** close up view on the cutting edge of Labrum, showing the teeth. Scale bars in µm.

####### Remarks.

The specimens examined in the present study agree with the description given by Chan (2009) and Chan et al. (2009). *Striatobalanus tenuis* closely resembles *S. amaryllis* (Darwin, 1854), but differs from the latter in the following characters: mandible with five equally spaced teeth (in *S. amaryllis*, the mandible has four teeth and the distance between the 3^rd^ and 4^th^ teeth is larger than the rest, Chan 2009); tergum triangular with short and wide spur (tergum narrow with beak produced apically in *S. amaryllis*, Chan 2009); and maxilla triangular, covered with dense setae (maxilla bilobed, elongated with dense setae on inferior margins in *S. amaryllis*, Chan 2009).

*Striatobalanus tenuis* also differs from *S. krugeri* (Pilsbry, 1916) and *S. taiwanensis* (Hiro, 1939) in that it has a median furrow on its tergum.

####### Worldwide distribution.

This species has been reported from South Africa, East China Sea, South China Sea, Japan, the Philippines, Indonesia (Chan 2009), Vietnam (Poltarukha 2010) and India (Krishnamoorthy 2007).

####### Distribution in India.

This species has been reported from Gujarat (present study), Tamil Nadu (Krishnamoorthy 2007; Daniel 1956), Odisha (formerly Orissa) (Nilsson-Cantell 1938), and Karnataka (Nilsson-Cantell 1938).

#### Balanidae Leach, 1817

##### *Amphibalanus* Pitombo, 2004

###### Amphibalanus amphitrite

Taxon classificationAnimaliaSessiliaBalanidae

(Darwin, 1854)

0229BB8E-F33D-52A7-8C1C-2F0C0FCEACE3

Figures 2F , 5

####### Examined material.

five specimens (BD: 8.29–17.16 mm), LFSc.ZRC-181, on fishing boat surface, Jakhau, Kachchh (23°11.30'N, 68°37.35'E), 21 August 2019, Gujarat, India, sandy shore, leg. M. Doshi.

####### Diagnosis

**Figure 5.**
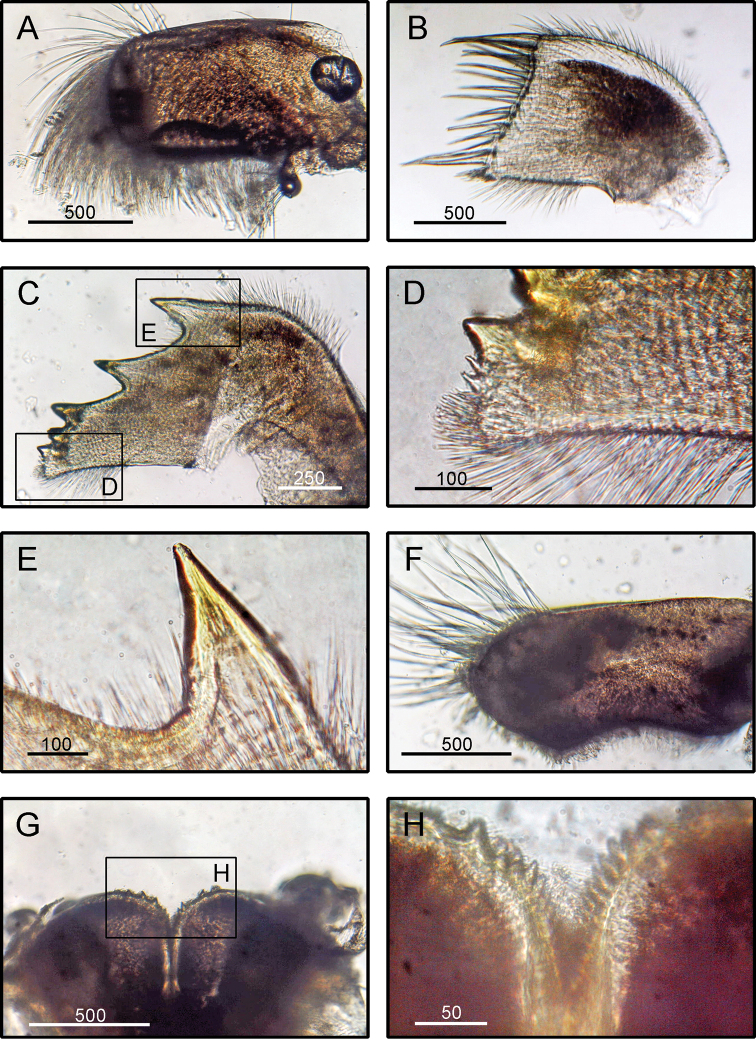
*Amphibalanus amphitrite* (Darwin, 1854), (BD: 12.38 mm) LFSc.ZRC-181, Light microscopy on mouth parts **A** maxilla **B** maxillule **C** mandible **D** close up on the inferior angle of mandible **E** close up on the teeth of mandible **F** mandibulatory palp **G** labrum **H** close up view on the cutting edge of Labrum, showing the teeth. Scale bars in µm.

**(modified from Chan et al. 2009).** Shell conical, outer surface smooth, with longitudinal deep-purple striations (Fig. 2F). No horizontal striations on shell surface. Tergum with short, wide spur (Fig. 5). Scutum usually flat, occasionally concave between the apex and the basal margin. Articular ridges prominent (Fig. 5). Maxilla bilobed with dense setae on all margins (Fig. 5A). Maxillule not notched, cutting edge straight, upper and lower margins bearing fine setae (Fig. 5B). Mandible with five teeth, upper three teeth sharp, well developed (Fig. 5C–E). Mandibulatory palp bearing setae on superior margin (Fig. 5F). Labrum with a deep cleft, ca. 13–22 teeth on each side of cutting edge (Fig. 5G, H).

####### Remarks.

The specimens examined in the present study agree with the descriptions given by Henry and McLaughlin (1975), Chan et al. (2009), and Pochai et al. (2017). *Amphibalanus amphitrite* closely resembles *A. reticulatus* (Utinomi, 1967) but differs from the latter in the following characters: the shell plates have only vertical purple striation (shell plates have longitudinal stripes intersected with transverse striations in *A. reticulatus*: Pochai et al. 2017), the shape of the shell is comparatively less columnar than in *A. reticulatus* (Pochai et al. 2017).

####### Worldwide distribution.

This species has been reported from Bermuda and southeast USA to Brazil, Hawaii, California to southwest Mexico, western European waters, Mediterranean Sea, south coast of Africa, Red Sea, Black Sea, Southeast Africa, India (Trivedi et al. 2015), Australia, Indonesia, Singapore, Malaysia, Gulf of Siam in Cambodia (Jones and Hosie 2016), Vietnam (Condor Islands, Tang Trien (South Annam), Cauda Nhatrang, Hongay, Tonkin), the South China Sea, Bohai Sea (China), Taiwan, the Philippines, Japan (South Honshu, Kyushu and Ryukyu Islands) and Vladivostok (Russia) (see review in Henry and McLaughlin (1975)).

####### Distribution in India.

This species has been reported from Gujarat (Trivedi et al. 2015; Parmar et al. 2018; present study), Maharashtra (Bhatt and Bal 1960), Goa (Desai et al. 2018), Kerala (Nilsson-Cantell 1938), Tamil Nadu (Prasanth and Sureshkumar 2020), Andhra Pradesh (Rao and Balaji 1988), Pulicat Lake (Sanjeeva 2006), Odisha (formerly Orissa) (Mitra et al. 2010), West Bengal (Ramakrishna and Talukdar 2003), and Andaman and Nicobar Islands (Mishra et al. 2010).

###### Amphibalanus reticulatus

Taxon classificationAnimaliaSessiliaBalanidae

(Utinomi, 1967)

9B61190F-5780-5720-BE3F-F560CC18DA79

Figures 2G , 6

####### Examined material.

Two specimens (BD: 14.99 mm and 14.35 mm), LFSc.ZRC-158, Jakhau, Kachchh (23°11.30'N, 68°37.35'E), 9 January 2020, Gujarat, India, rock surface, leg. M. Doshi.

####### Diagnosis

**Figure 6.**
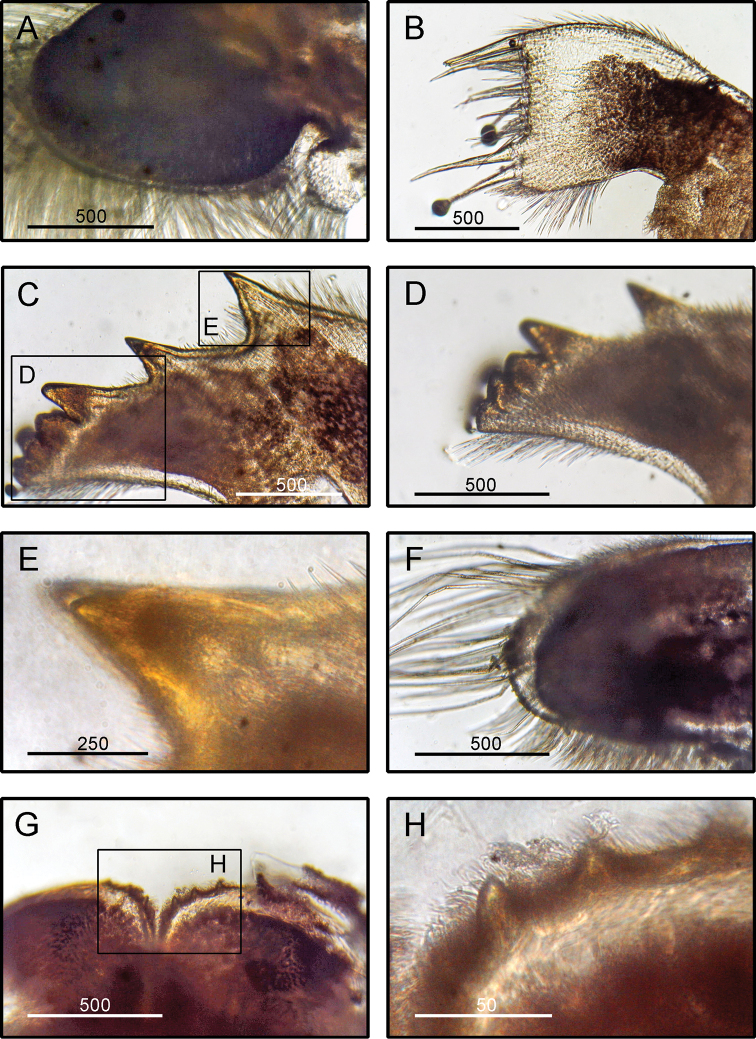
*Amphibalanus reticulatus* (Utinomi, 1967), (BD: 14.99 mm), LFSc.ZRC-158, light microscopy on mouth parts **A** maxilla **B** maxillule **C** mandible **D** close up on the inferior angle of mandible **E** close up on the teeth of mandible **F** mandibulatory palp **G** labrum **H** close up view on the cutting edge of labrum, showing the teeth. Scale bars in µm.

**(modified from Chan et al. 2009).** Shell conical surface smooth, having purple, pink, and white longitudinal stripes which intersect with transverse striations, operculum diamond-shaped (Fig. 2G). Scutum triangular with scutal margin straight (Fig. 2G). Tergum with straight occludent margins and short spur (Fig. 2G). Maxilla bilobed with margins bearing dense setae (Fig. 6A). Maxillule not notched (Fig. 6B). Mandible with four teeth excluding inferior, inferior angle blunt, 4^th^ teeth bidentate (Fig. 6C–E). Mandibulatory palp with setae only on superior margin (Fig. 6F). Labrum with a deep cleft and four teeth on each side of cutting edge (Fig. 6G, H).

####### Remarks.

The specimens examined in the present study agree with the descriptions and illustrations given by Chan et al. (2009) and Pochai et al. (2017). *Amphibalanus reticulatus* is very similar to *A. variegatus* (Darwin, 1854), in which both shells have striated patterns. Pitriana et al. (2020) illustrated the scutum, tergum, and mandibles of *A. variegatus*. The gaps between the teeth in the mandibles are smaller in *A. variegatus* than in *A. reticulatus*. In the present study, we concluded the mandibles of the specimens collected from India have relatively larger gaps between the teeth compared to the illustration in Pitriana et al. (2020). In addition, the tergum of *A. variegatus* illustrated in Pitriana et al. (2020) has a sharp spur, while the spur of the Indian specimen is blunt. We conclude the specimens collected in the present study represent *A. reticulatus*.

####### Worldwide distribution.

This species has been reported from Japan, Indo-West Pacific (the Philippines, Hawaii, Gulf of Thailand, Indonesia; Chan et al. 2009; Pochai et al. 2017), Australia, Persian Gulf, and India (Fernando 2006).

####### Distribution in India.

This species has been reported from Gujarat (present study), Maharashtra (Swami et al. 2011), and Tamil Nadu (Fernando 2006).

##### *Megabalanus* Hoek, 1913

###### Megabalanus tintinnabulum

Taxon classificationAnimaliaSessiliaBalanidae

(Linnaeus, 1758)

0443F577-0DC0-50D7-B078-FA9995B6BB3A

Figures 2D, E , 7

####### Examined material.

Five specimens (BD: 10.57–24.26 mm), LFSc.ZRC-182, Veraval, Gir Somnath district (20°54.60'N, 70°21.13'E), 18 November 2019, Gujarat, India, rocky shore, leg. K. Patel.

####### Diagnosis

**Figure 7.**
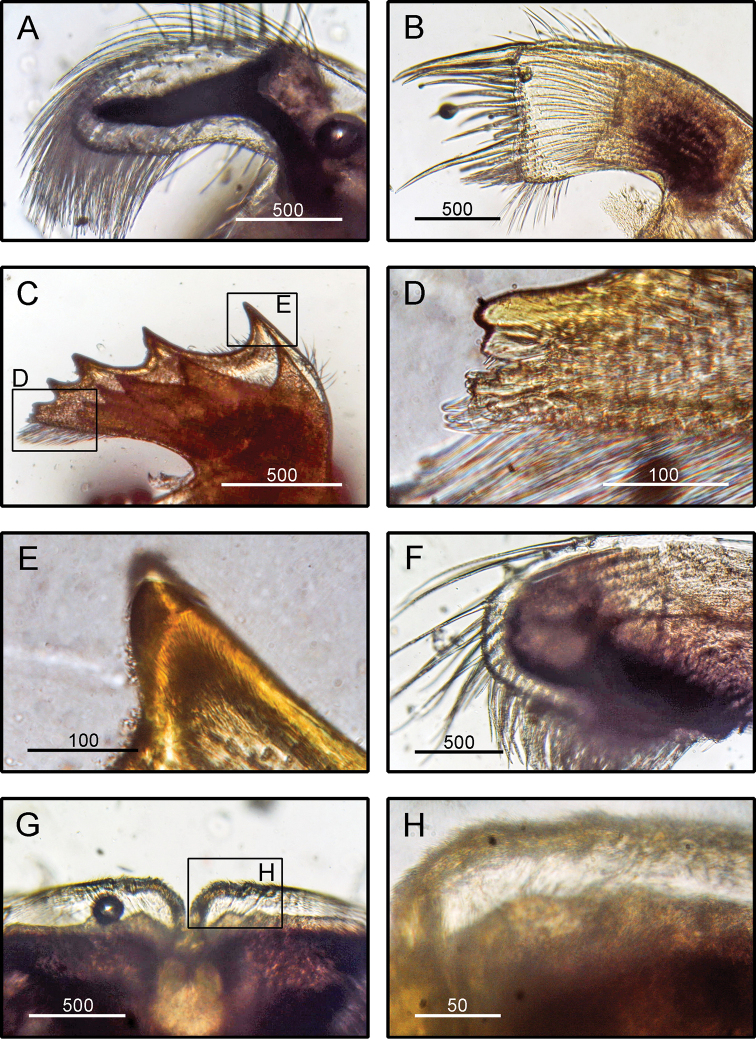
*Megabalanus tintinnabulum* (Linnaeus, 1758), (BD: 20.28 mm) LFSc.ZRC-182, Light microscopy on mouth parts **A** maxilla **B** maxillule **C** mandible **D** close up on the inferior angle of mandible **E** close up on the teeth of mandible **F** mandibulatory palp **G** labrum **H** close up view on the cutting edge of labrum, showing the teeth. Scale bars in µm.

**(modified from Chan et al. 2009).** Shell cylindrical to conical, colouration variable, mostly with rosy pink longitudinal stripes, surface smooth (Fig. 2D). Scutum triangular, with prominent transverse growth ridges, external surface bearing horizontal striations, inner surface with conspicuous articular ridges, articular ridges broad (Fig. 2E). Tergum broad and triangular, spur long, narrow, prominent. External surface with median furrow (Fig. 2E). Maxilla bilobed with setae on all margins (Fig. 7A). Maxillule not notched, cutting edge straight (Fig. 7B). Mandible with 5 teeth excluding inferior angle, 1^st^ tooth largest, sharply pointed, inferior angle blunt (Fig. 7C–E). Mandibulatory palp rectangular, with setae on superior margin (Fig. 7F). Labrum with very hairy crest and a deep cleft (Fig. 7G, H).

####### Remarks.

The specimens examined in the present study agree with the original description given by Linnaeus (1758) and the more recent one by Chan et al. (2009). However, in the present specimen, the labrum does not possess teeth whereas the specimen examined by Chan et al. (2009) has three sharp teeth on each side of the cutting edge.

*Megabalanus tintinnabulum* closely resembles *M. validus* Darwin, 1854, but differs from the latter in having a conical shell with a coloured external surface. The species also resembles *M. volcano* (Pilsbry, 1916), but differs from the latter in having the maxillule not notched.

####### Worldwide distribution.

The species has a cosmopolitan distribution with records from Brazil, Venezuela, European waters (UK, Ireland, Belgium and Netherlands; Southward, 2008) the Mediterranean Sea, Madagascar, Cape of Good Hope, New Zealand, Australia, Singapore, Thailand, Vietnam (Jones and Hosie 2016), Hong Kong, Taiwan, Japan, and India (Trivedi et al. 2015).

####### Distribution in India.

This species has been reported from Gujarat (Trivedi et al. 2015; Parmar et al. 2018; present study), Maharashtra (Karande and Palekar 1966), Goa (Nandakumar 1990), Tamil Nadu (Krishnamoorthy 2007), Andhra Pradesh (Rao and Balaji 1988), Odisha (formerly Orissa) (Pati et al. 2009), West Bengal (Nilsson-Cantell 1938), Andaman and Nicobar Islands (Daniel 1972), and the Bay of Bengal (Nilsson-Cantell 1938).

#### Coronuloidea Leach, 1817

**Chelonibiidae Pilsbry, 1916**

##### *Chelonibia* Leach, 1817

###### Chelonibia testudinaria

Taxon classificationAnimaliaSessiliaChelonibiidae

(Linnaeus, 1758)

DB0EAC8F-0D68-5F38-A56A-42153A8DD12B

Figures 2H, I , 8

####### Examined material.

Two specimens (BD 5.33 and 5.59 mm), LFSc.ZRC-159, on carapace of crab *Portunus sanguinolentus*, Kuda, Bhavnagar (21°37.70'N, 72°18.40'E), 17 April 2019, Gujarat, India, sandy shore, leg. J. Trivedi.

####### Diagnosis.

**Figure 8.**
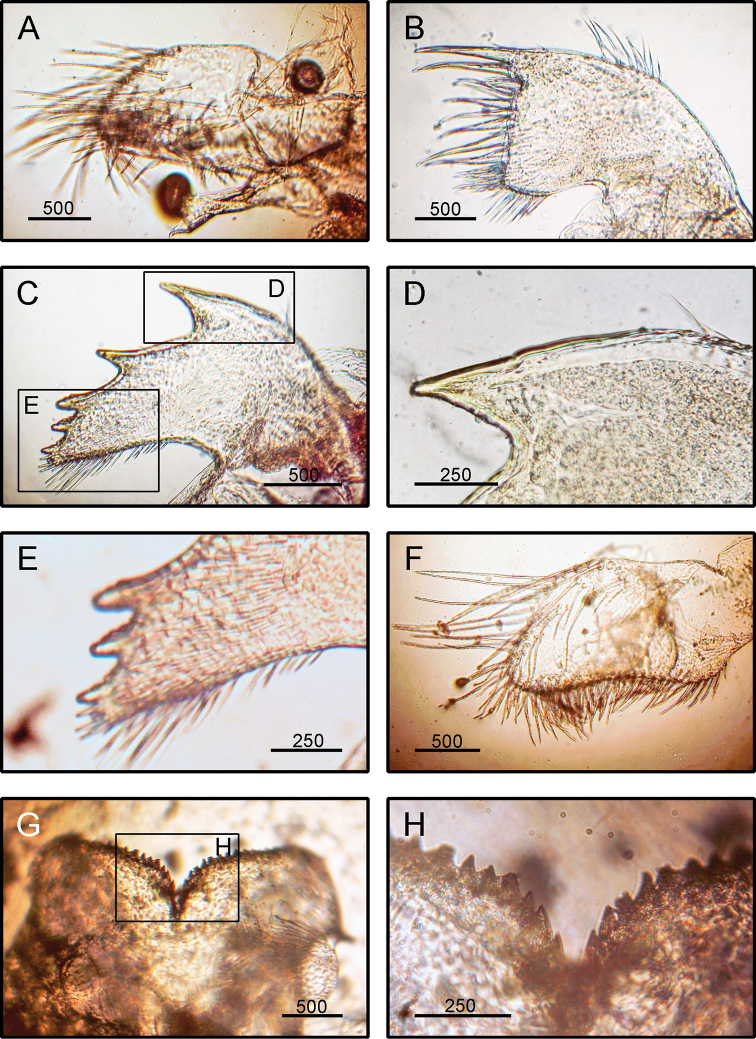
*Chelonibia testudinaria* (Linnaeus, 1758), (BD: 5.33 mm) LFSc.ZRC-159, Light Microscopy on mouth parts **A** maxilla **B** maxillule **C** mandible **D** close up on the inferior angle of mandible **E** close up on the teeth of mandible **F** mandibulatory palp **G** labrum **H** close up view on the cutting edge of labrum, showing the teeth. Scale bars in µm.

Shell white, slightly conical and six-plated, radii board. Specimens living on turtles display oval-shaped depressions on radii of each shell plate. Specimens living on surfaces of decapods have a smooth outer surface, without any depressions on radii (Fig. 2I). Aperture large, scutum and tergum reduced, elongated rectangular in shape (Fig. 2I). Maxilla bilobed (Fig. 8A); maxillule feebly notched, cutting edge straight (Fig. 8B); mandible with five teeth, lower margin short (Fig. 8C–E). Mandibulatory palp elongated with rough edges (Fig. 8F). Labrum having cleft with numerous sharp teeth (Fig. 8G, H).

####### Remarks.

Previously, *Chelonibia* living on decapods were identified as *C. patula* and *Chelonibia* living on sea turtles as *C. testudinaria*. Cheang et al. (2013) and Zardus et al. (2014) revealed there is no significant genetic difference between *C. patula* and *C. testudinaria*, suggesting that these are the same species and their morphological differences are the result of phenotypic plasticity. We consider *C. testudinaria* as including two major morphs. The *patula* morph has a smooth white shell and lives mainly on decapods, while the *testudinaria* morph has oval depressions on the radii and lives mainly on surfaces of turtles. Dwarf males are often housed in these depressions on the *testudinaria* morph (Zardus and Hadfield 2004; Collareta 2020).

####### Worldwide distribution.

*Chelonibia testudinaria* has been recorded in the Atlantic Ocean, Pacific Ocean and the Mediterranean Sea (Pasternak et al. 2002; Rawson et al. 2003) including Greece (Kitsos et al. 2003, 2005), Israel (Pasternak et al. 2002), Italy (Relini 1980; Frazier and Margaritoulis 1990), and Turkey (Bakir et al. 2010). Further records included Australia (Jones and Hosie 2016), Pakistan (Javed and Mustaquim 1994), and India (Krishnamoorthy 2007).

####### Distribution in India.

This species has been reported from Gujarat (Frazier 1990; present study), Maharashtra (Wagh and Bal 1974), Kerala (Pillai 1958), Lakshadweep Islands (Hayashi 2013), Tamil Nadu (Daniel 1956; Krishnamoorthy 2007), Andhra Pradesh (Nilsson-Cantell 1938), Pulicat lake (Daniel 1981), Odisha (formerly Orissa) (Nilsson-Cantell 1938), west Bengal (Daniel 1981), and Andaman and Nicobar Islands (Nilsson-Cantell 1938).

#### Tetraclitoidea Gruvel, 1903

**Tetraclitidae Gruvel, 1903**

##### *Tetraclita* Schumacher, 1817

###### Tetraclita ehsani

Taxon classificationAnimaliaSessiliaTetraclitidae

Shahdadi, Chan & Sari, 2011

9EFFF361-76C6-5187-8B4F-5CB24EDB5C03

Figures 3A, B , 9

####### Examined material.

Five specimens (BD: 8.37–16.58 mm), LFSc.ZRC-184, Sutrapada, Gir Somnath district (20°50'23"N, 70°28'28"E), 22 December 2019, Gujarat, India, rocky shore, leg. K. Patel.

####### Diagnosis

**Figure 9.**
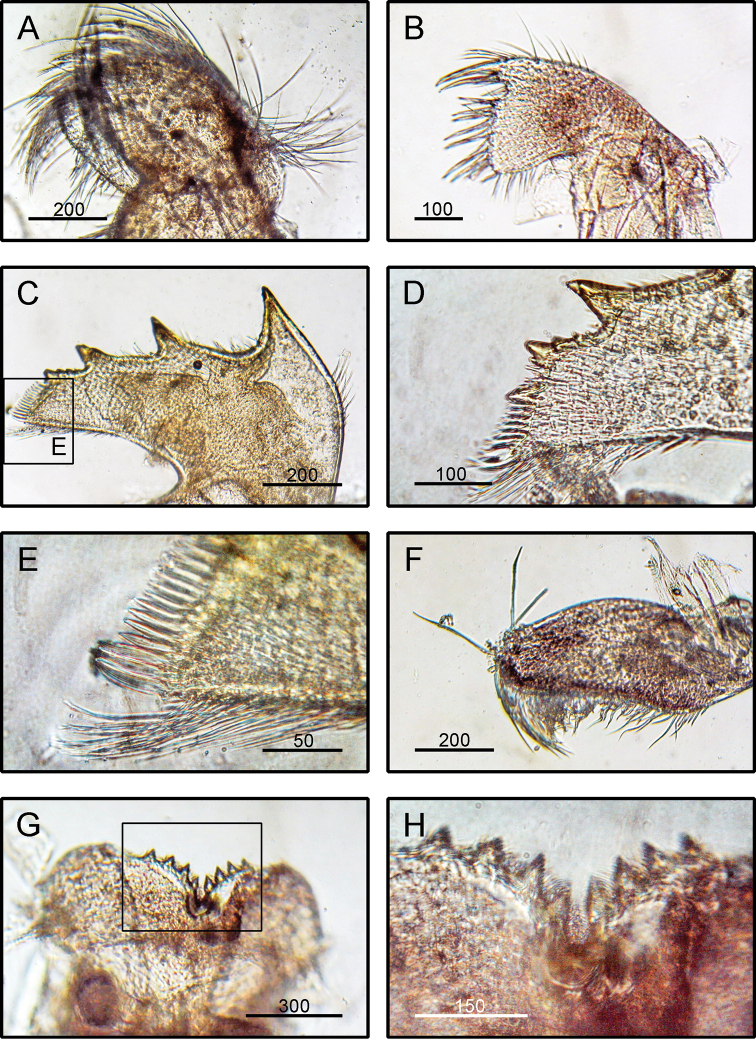
*Tetraclita ehsani* Shahdadi, Chan & Sari, 2010, (BD: 14.38 mm), LFSc.ZRC-184 Light microscopy on mouth parts **A** maxilla **B** maxillule **C** mandible **D** close up on the inferior angle of mandible **E** close up on the teeth of mandible **F** Mandibulatory palp **G** labrum **H** close up view on the cutting edge of labrum, showing the teeth. Scale bars in µm.

**(modified from Shahdadi et al. 2011).** Shell four-plated, conical, pink (Fig. 3A). Scutum and tergum white. Scutum narrow, external surface bearing faint horizontal striations, 1.5 × higher than wide, adductor muscle pit shallow, seven distinct rostral and four–six lateral depressor crests (Fig. 3B). Tergum long and narrow with ten definite depressor crests, spur long and narrow (Fig. 3B). Maxilla bilobed and setae present on both the lobes (Fig. 9A). Maxillule notched with two large and four small simple setae above notch (Fig. 9B). Mandible with five teeth excluding the inferior angle, 1^st^ tooth separated from the remaining teeth, 2^nd^ and 4^th^ teeth bidentate, 3^rd^ teeth tridentate, 5^th^ tooth small and located close to the 4^th^ tooth (Fig. 9C–E). Mandibulatory palps elongated, superior margin bearing setae (Fig. 9F). Labrum notched, notch shallow, four erect large teeth on each side of the cutting edge (Fig. 9G, H).

####### Remarks.

The examined specimens in the present study agree with the description given by Shahdadi et al. (2011). *Tetraclita ehsani* closely resembles *T. reni* Chan, Hsu & Tsai, 2009, *T. achituvi* Ross, 1999 and *T. rufotincta* Pilsbry, 1916, but can be differentiated from these species in the following characters: the tergum is very narrow, with the basal region slightly concave or almost straight vs. the broad tergum that has a strongly concave basal margin in *T. rufotincta* and *T. reni*, and the basi-carinal angle is larger (~ 100°) (the basi-carinal angle is smaller in *T. reni* (80°) and *T. rufotincta* (73°) (Shahdadi et al. 2011).

####### Worldwide distribution.

This species has been reported from the Gulf of Oman in Iran (Shahdadi et al. 2011) and from northwest India (Tsang et al. 2012).

####### Distribution in India.

This species has been reported from Gujarat (Tsang et al. 2012; present study). It is not found in the region further south of Gujarat and was confirmed to be absent in Mumbai and southern India (Tsang et al. 2012).

##### *Tetraclitella* Hiro, 1939

###### Tetraclitella karandei

Taxon classificationAnimaliaSessiliaTetraclitidae

Ross, 1971

FB011EE1-0C7C-54A0-8ED2-530E3570B5A2

Figures 3C–E , 10

####### Examined material.

Five specimens (BD: 5–10 mm), ASIZCR000454, Nagoa Beach, Diu (20°42.12'N, 70°55.02'E), 22 March 2010, Gujarat, India, rocky shore, leg. B.K.K. Chan.

####### Diagnosis.

**Figure 10.**
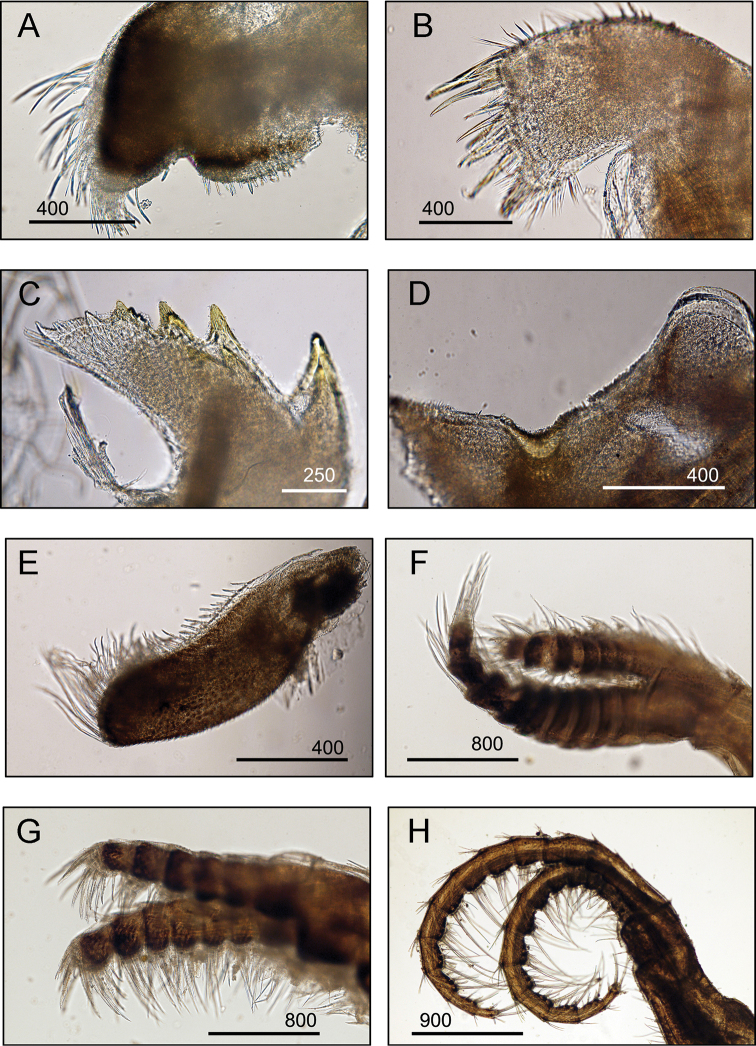
*Tetraclitella karandei* Ross, 1971, (BD: 8.37 mm), ASIZCR000454, Light microscopy on mouth parts **A** maxilla **B** maxillule **C** mandible **D** labrum **E** mandibulatory palp **F** cirrus I **G** cirrus II **H** cirrus III. Scale bars in µm.

Shell four-plated, surface of radii protruding with digit-like horizontal striations up to the shell apex, shell surface with fine hairs and chitin coating (Fig. 3C, D). Opercular plates white, scuta triangular, occludent margin and basal margin almost perpendicular, tergal margin straight; tergum higher than wide, scutal margin straight, spur small (Fig. 3E). Maxilla bilobed (Fig. 10A). Maxillule notched, with two cuspidate setae above notch (Fig. 10B). Mandible having four teeth, the 3^rd^ and 4^th^ of which are triple-dentated (Fig. 10C). Labrum slightly bullate, with two small teeth on each cutting edge (Fig. 10D). Mandibulatory palp elongated with dense setae on superior angle (Fig. 10E). Cirrus I: anterior ramus seven-segmented, posterior ramus longer and slender, nine-segmented. Cirrus II: rami subequal, anterior ramus six-segmented, posterior ramus seven-segmented (Fig. 10F–H). Cirrus III: both rami slender, anterior ramus 13-segmented, posterior ramus 14-segmented. Intermediate segment bears two pairs of long simple setae and three pairs of short simple setae (Fig. 10F–H).

####### Remarks.

This species inhabits intertidal shore of the rocky intertidal region of Gujarat.

####### Worldwide distribution.

This species has been recorded in India and Taiwan (Ross 1971, 1972).

####### Distribution in India.

This species has been reported from Gujarat (present study) and Mumbai (Ross 1971; Fernando 2006).

#### Chthamaloidea Darwin, 1854

**Chthamalidae Darwin, 1854**

##### *Chthamalus* Ranzani, 1817

###### Chthamalus barnesi

Taxon classificationAnimaliaSessiliaChthamalidae

Achituv & Safriel, 1980

D6B38229-4C1A-5E19-A025-6C3021BD42FC

Figures 3F–H , 11

####### Examined material.

Five specimens (BD: 3.03–5.57 mm), LFSc.ZRC-160, Shivrajpur, Jamnagar District (22°20'03"N, 68°57'17"E), 17 February 2019, Gujarat, India, rocky shore, leg. M. Doshi.

####### Diagnosis

**Figure 11.**
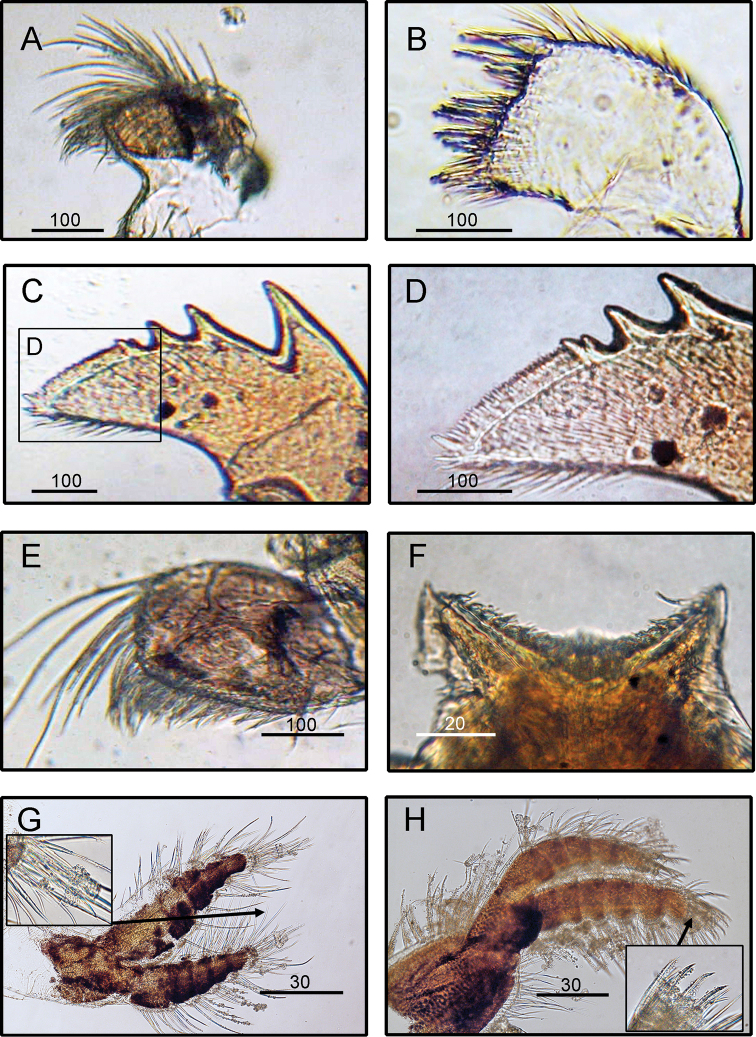
*Chthamalus barnesi* Achituv & Safriel, 1980. (BD: 4.21 mm), LFSc.ZRC-160, Light microscopy on mouth parts **A** maxilla **B** maxillule **C** mandible **D** close up on the inferior angle of mandible **E** mandibulatory palp **F** labrum **G** cirrus II **H** cirrus III. Scale bars in µm.

**(modified from Shahdadi and Sari 2011).** Shell orifices almost kite-shaped (Fig. 3F, G). Tergum narrow with upper part broader than lower part and suture between tergum and scutum zigzag-shaped (Fig. 3H). Scutum elongated and triangular and lateral depressor muscle pit distinct without crest (Fig. 3H). Maxilla bilobed (Fig. 11A). Maxillule not notched or possess very shallow notch (Fig. 11B). Lower part of maxillule is setose. Mandible with four teeth (Fig. 11C). Basal comb with rows of 16–23 short spines and 2–4 stout large spines at lower angle (Fig. 11D). Mandibulatory palp with dense setae on all margins (Fig. 11E). Labrum with numerous fine teeth present (Fig. 11F). Cirrus I: anterior ramus (with seven or eight segments) longer than posterior (usually with 5–7 segments) (Fig. 11G). Cirrus II: anterior ramus (with six–seven segments) longer than posterior (usually with 5–7 segments) (Fig. 11H). Cirri III–VI: rami almost equal in size.

####### Remarks.

The examined specimen in the present study agree with the description given by Achituv and Safriel (1980) and Shahdadi and Sari (2011). *Chthamalus barnesi* forms part of the *challengeri* group and closely resembles *C. moro* Pilsbry, 1916, *C. neglectus* Yan & Chan, 2004, and *C. challengeri* Hoek, 1883, but can be differentiated based on the following characters: a depression towards the tergo-occludent corner of the scutum (*C. moro*, lacks this depression, Southward and Newman 2003), the tergal margin is not straight (tergal margin straight in *C. moro*, Southward & Newman, 2003), the scutal margin of the tergum shows a deep articular furrow (scutal margin of tergum almost straight in *C. neglectus*, Yan & Chan, 2004), and the maxillule possess a very shallow notch (maxillule possesses a distinct notch in *C. challengeri*, Shahdadi & Sari, 2011).

####### Worldwide distribution.

The species has been reported from the Red Sea, Gulf of Aden, and Gulf of Oman including Yemen, Oman, Iran, Saudi Arabia (Shahdadi and Sari 2011), and northwest India (present study).

####### Distribution in India.

This species is reported for the first time in India from the coastal regions of Gujarat.

##### *Microeuraphia* Poltarukha, 1997

###### Microeuraphia withersi

Taxon classificationAnimaliaSessiliaChthamalidae

(Pilsbry, 1916)

849F9E65-FD1D-5AA4-9BA6-B726E75F1D7D

Figures 3I, J , 12

####### Examined material.

Five specimens (BD: 3.90–6.01 mm) LFSc.ZRC-161, Kuda, Bhavnagar (21°37.70'N, 72°18.40'E), 21 January 2020, Gujarat, India, muddy shore, leg. M. Doshi.

####### Diagnosis

**Figure 12.**
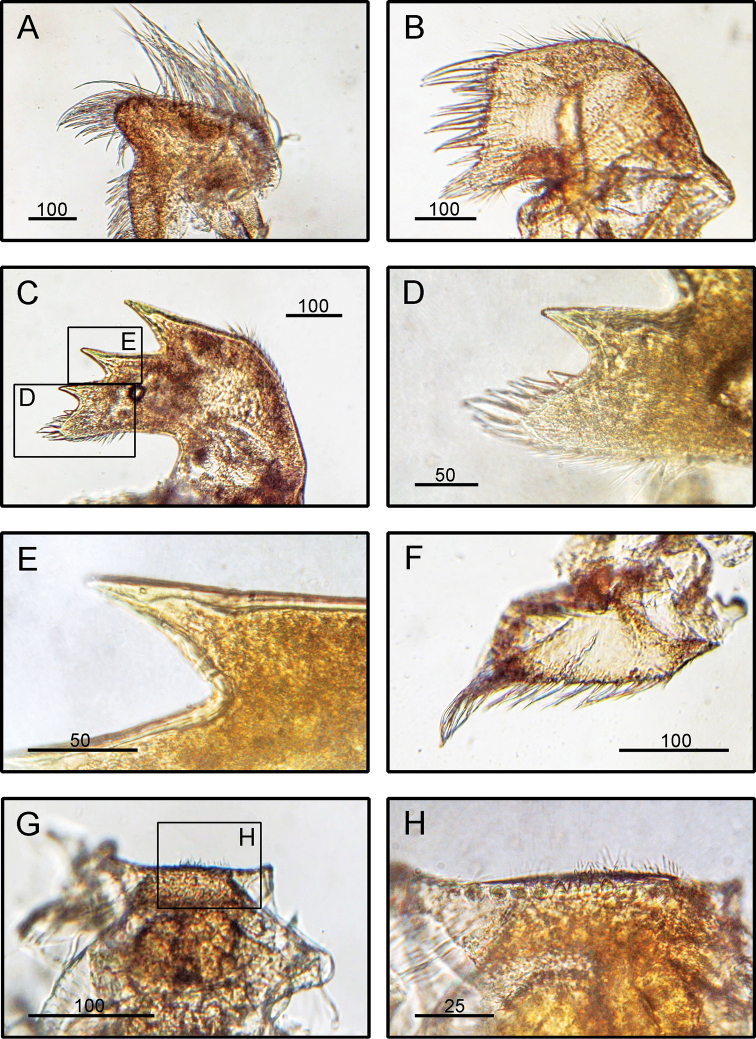
*Microeuraphia withersi* (Pilsbry, 1916). (BD: 6.01 mm), LFSc.ZRC-161, Light microscopy on mouth parts **A** maxilla **B** maxillule **C** mandible **D** close up on the inferior angle of mandible **E** close up on the teeth of mandible **F** mandibulatory palp **G** labrum **H** close up view on the cutting edge of labrum, showing the teeth. Scale bars in µm.

**(modified from Pilsbry 1916).** Specimens depressed, cinnamon-brown with smooth surface, with a large, wide aperture; alae broad with arched, sub-horizontal summits (Fig. 3I). Scutum thin, triangular, conical, almost twice as long as wide, lower part with fine growth-lines (Fig. 3J). Articular ridge feebly developed with median lobe, not extending beyond the scutal border. Articular furrow shallow and sharply notched. Tergum narrow, club-shaped, very thick (Fig. 3J). Cirrus I: anterior ramus (with seven or eight segments) longer than posterior (usually with six or seven segments). Cirrus II: anterior ramus (with seven or eight segments) longer than posterior (usually with six segments). Setae of terminal segment non-pectinated. The carinal lobe narrow, situated high. Maxilla bilobed (Fig. 12A), group of short spines on the lower edge. Maxillule not notched (Fig. 12B). Mandible with three large teeth and pectinated lower point with eight spines (Fig. 12C–E). Mandibulatory palp rectangular (Fig. 12F). Labrum with broad, nearly straight edge, the middle fold having a series of strong teeth (Fig. 12G, H).

####### Remarks.

The specimens examined in the present study agree with the description by Pilsbry (1916). *Microeuraphia withersi* closely resembles *M. depressa* and *M. permitini*, but can be distinguished from the latter based on the following characters: the scutum is comparatively narrow in (scutum is comparatively wide in *M. depressa*, Poltarukha, 1997), the width to height ratio fluctuates from 0.8 to 1.4 (width to height ratio commonly > 1.5 in *M. depressa*, Poltarukha, 1997), the basal comb of mandible with eight equally distanced slender spines (1–3 stout spines after third tooth, and a row of small and 2–4 long spines in *M. permitini*, Shahdadi and Sari 2011), and both the rami of cirri II without finely pectinate setae on terminal segments (both rami of cirri II with finely pectinate setae on terminal segments in *M. permitini*; Shahdadi & Sari, 2011).

####### Worldwide distribution.

The species has been reported from the Philippines (Pilsbry 1916), the west coast of Sumatra (Nilsson-Cantell 1921), Indonesia, Singapore, Java, Vietnam, Hong Kong, the South China Sea (Jones and Hosie 2016), the East China Sea (Zevina and Tarasov 1963), Australia, Madagascar (Utinomi 1968), and India (Nilsson-Cantell 1938).

####### Distribution in India.

This species is reported from Gujarat (present study), Maharashtra (Nilsson-Cantell 1938; Karande and Palekkar 1966; Wagh and Bal 1974), and West Bengal (Daniel 1981).

#### Lepadiformes Buckeridge & Newman, 2006

**Lepadidae Darwin, 1852**

##### *Lepas* Linnaeus, 1758

###### Lepas anatifera

Taxon classificationAnimaliaPedunculataLepadidae

Linnaeus, 1758

67A257A1-7EBD-521B-821A-91C00ABE44EA

Figures 2J , 13

####### Examined material.

Five specimens (CL: 8.29–16.39 mm), LFSc.ZRC-162, Jakhau, Kachchh (23°11.30'N, 68°37.35'E), 26 July 2019, Gujarat, India, fishing boat surface, leg. M. Doshi.

####### Diagnosis

**Figure 13.**
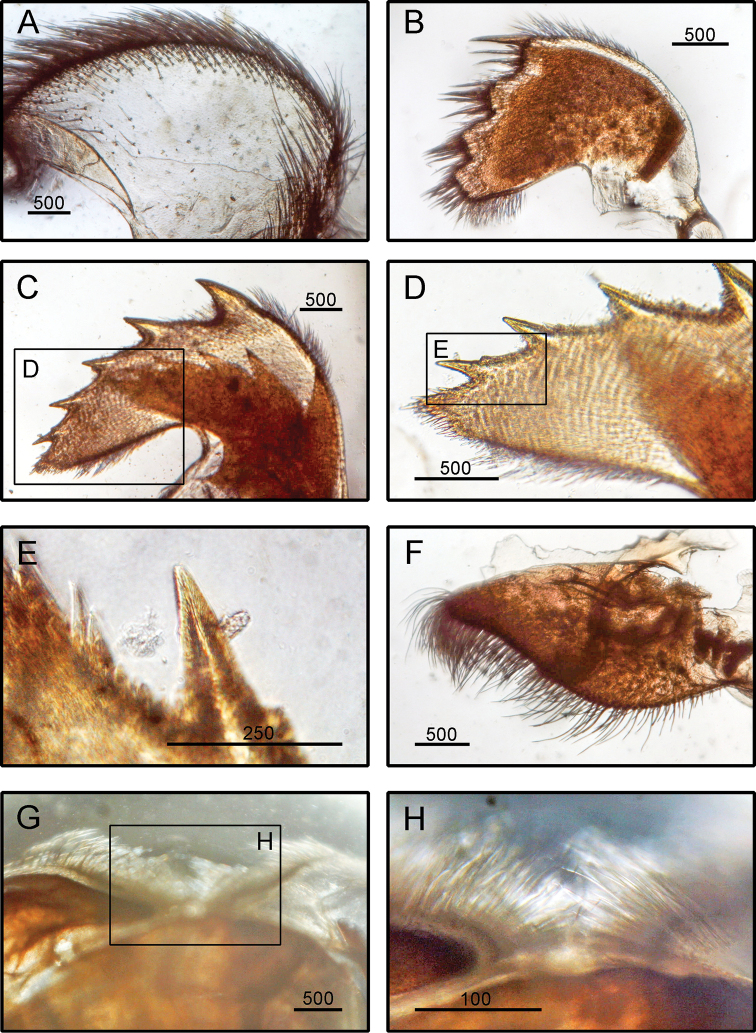
*Lepas anatifera* Linnaeus, 1758 (CL: 16.39 mm) LFSc.ZRC-162, Light microscopy on mouth parts **A** maxilla **B** maxillule **C** mandible **D** close up on the inferior angle of mandible **E** close up on the teeth of mandible **F** mandibulatory palp **G** labrum **H** close up view on the cutting edge of labrum, showing the teeth. Scale bars in µm.

**(modified from Chan et al. 2009).** Capitulum with five smooth, white, thin plates. Capitulum white, peduncle dark brown in colour (Fig. 2J). Scutum triangular with occludent margin convex. Right scutum with inner umbonal tooth, sometimes rudimentary. Scutum sometimes with dark marking or spots, carina branched below umbo. Tergum triangular to quadrangular with occludent margin convex or angular, apex almost truncated. Carina generally smooth, occasionally barbed. Peduncle variable in length, sometimes several times longer than capitulum. Caudal appendages short and claw-shaped. Maxilla globular with setae over margins (Fig. 13A). Maxillule notched into four distinct regions (Fig. 13B). Mandible having five teeth excluding inferior angle, inferior angle pectinated (Fig. 12C–E). Mandibulatory palp triangular with setae on superior margin (Fig. 12F). Labrum prominently concave, fine setae and teeth on cutting edge (Fig. 12G, H).

####### Remarks.

The specimens examined in the present study agree with the description given by Chan et al. (2009). *Lepas anatifera* closely resembles *L. anserifera* Linnaeus, 1767, but can be differentiated by the following characters: maxillule notched into four distinct regions (maxillule not clearly notched in *L. anserifera*, Chan et al. 2009), upper portion of tergum blunt (upper portion of tergum pointed in *L. anserifera*, Chan et al. 2009), scutum sometimes with dark marking or spots (no such markings or spots on scutum in *L. anserifera*, Chan et al. 2009).

####### Worldwide distribution.

The species has a cosmopolitan distribution (Chan et al. 2009; Schiffer and Herbig 2016) that includes India (Krishnamoorthy 2007).

####### Distribution in India.

This species has been reported from Gujarat (present study), Tamil Nadu (Krishnamoorthy 2007), Odisha (formerly Orissa) (Annandale 1909; Ramakrishna and Talukdar 2003), and Andaman and Nicobar Islands (Nilsson-Cantell 1938).

###### Lepas anserifera

Taxon classificationAnimaliaPedunculataLepadidae

Linnaeus, 1767

59D63014-4C28-5546-BFA0-E62533FD0F83

Figures 2K , 14

####### Examined material.

Five specimens (CL: 11.39–22.13 mm), LFSc.ZRC-163, Vankbara beach, Diu (20°42.88'N, 70°53.16'E), 12 December 2019, Gujarat, India, fishing boat surface, leg. M. Doshi.

####### Diagnosis

**Figure 14.**
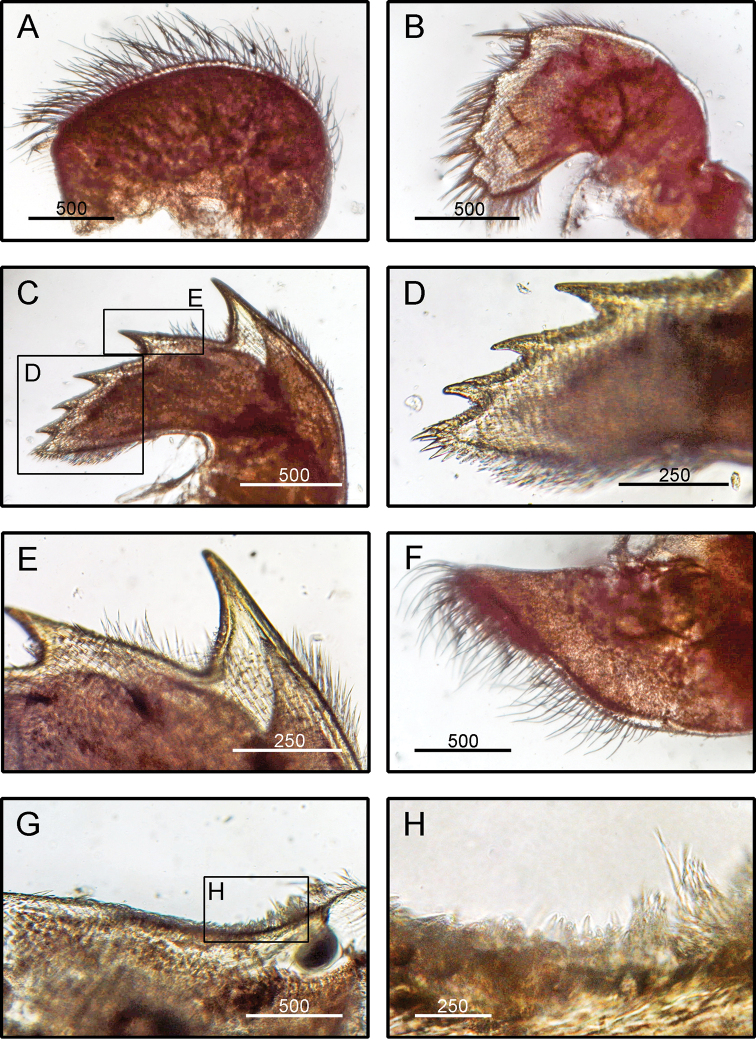
*Lepas anserifera* Linnaeus, 1758, (CL: 16.28 mm) LFSc.ZRC-183, Light microscopy on mouth parts **A** maxilla **B** maxillule **C** mandible **D** close up on the inferior angle of mandible **E** close up on the teeth of mandible **F** mandibulatory palp **G** labrum **H** close up view on the cutting edge of labrum, showing the teeth. Scale bars in µm.

**(modified from Chan et al. 2009).** Capitulum five-plated, plates thick, broadly triangular, slightly compressed, white, surface striated with radiating lines (Fig. 2K). Tergum quadrilateral, wider than high, apex beaked but sometimes rounded off. Scutum fan-shaped, occludent margin strongly convex. Carina forked, produced below the base of scutum. Maxilla globular (Fig. 14A). Maxillule not clearly notched, cutting edge with several dense setal aggregations (Fig. 14B). Mandible with five teeth excluding inferior teeth, lower angle pectinate (Fig. 14C–E). Mandibulatory palp triangular, setae present on inferior margin (Fig. 14F). Labrum concave bearing fine teeth (Fig. 14G, H).

####### Remarks.

The specimens examined in the present study agree with the descriptions given by Fernando (2006) and Chan et al. (2009).

####### Worldwide distribution.

This species has a cosmopolitan distribution in tropical and temperate seas (Chan et al. 2009; Jones and Hosie 2016; Schiffer and Herbig 2016) and in India (Annandale 1909).

####### Distribution in India.

This species has been reported from Gujarat (Parmar et al. 2018; present study), Tamil Nadu (Sundararaj 1927), Andhra Pradesh (Nilsson-Cantell 1938), Odisha (formerly Orissa) (Annandale 1909), West Bengal (Annandale 1909), and Andaman and Nicobar Islands (Annandale 1909).

### Zonation patterns of rocky intertidal species

**Figure 15.**
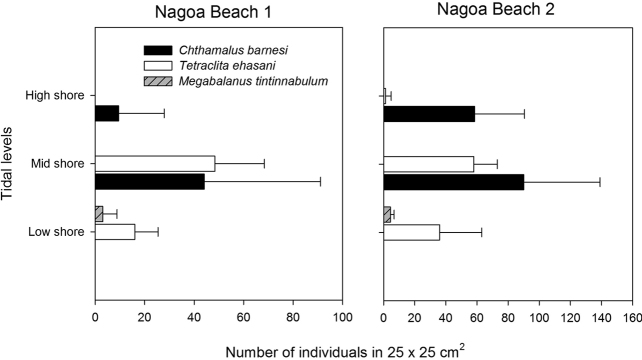
Mean (+1 SD, n = 10) density of barnacles on two rocky shores in Diu, Gujarat, India. High shore – 2 metres above C.D. Mid shore – 1.5 metres above C.D. Low shores 1 metre above C.D.

The high shores (2 m above C.D.) of the sandstone rocky shores at Diu are filled with *Chthamalus barnesi*, reaching a mean abundance of 20–50 individuals per 0.25 × 0.25 m^2^ quadrat. In the mid-shores (1.5 m above C.D.), *C. barnesi* and *T. ehsani* occur together, with similar abundances of 40–90 individuals per 0.25 × 0.25 m^2^. In the low shores, *C. barnesi* is absent, and *T. ehsani* has a low abundance and co-exists with *Megabalanus tintinnabulum* (Fig. 15).

### Key to barnacle species in Gujarat

**Table:** 

1	Without a stalk	**2**
–	With a stalk	**10**
2	Shell six-plated	**3**
–	Shell four-plated	**9**
3	Shell surface with longitudinal purple stripes	***Amphibalanus amphitrite***
–	Shell without longitudinal stripes	**4**
4	Shell with striated lattice pattern	***Amphibalanus reticulatus***
–	Shell without striated lattice pattern	**5**
5	Base calcareous	**6**
–	Base membranous	**8**
6	Shell with very wide radii	***Megabalanus tintinnabulum***
–	Shell without wide radii	***Striatobalanus tenuis***
7	Scutum and tergum reduced–	***Chelonibia testudinaria***
–	Scutum and tergum not reduced	**9**
8	Mandible four-toothed	***Chthamalus barnesi***
–	Mandible three-toothed	***Microeuraphia withersi***
9	Shell without distinct radii	***Tetraclita ehsani***
–	Shell with wide radii, surface with digit-like patterns	***Tetraclitella karandei***
10	Tergum without a sharp beak	***Lepas anatifera***
–	Tergum with a sharp beak	***Lepas anserifera***

## Discussion

The present study reported a total of eleven species from Gujarat, northwest India and is the first record of the rocky intertidal barnacle *Chthamalus barnesi* in India. *Tetraclita ehsani* was previously recorded from the Gulf of Oman, Iran, and northwest India. *Tetraclita ehsani* is absent from the Persian Gulf and Red Sea, where *T. rufotincta* is a common species (Tsang et al. 2012). Northwest India is probably the southern limit of *T. ehsani*, as this species is absent from Mumbai and Tamil Nadu (based on personal sampling trips by BKKC). *Chthamalus barnesi* was first identified along the coastline of the inner Red Sea (Achituv and Safriel 1980) and was subsequently reported in the Persian Gulf and Gulf of Oman (Shahdadi et al. 2011). Northwest India appears to be the eastern biogeographical limit of *C. barnesi*, as it is absent from Mumbai and further south. From Mumbai and along the southern and eastern coastlines of India, *C. malayensis* becomes dominant (Tsang et al. 2012). Based on the classification of the world’s biogeographical provinces and ecoregions by Spalding et al. (2007), Gujarat is located in the Western India Ecoregion of the West and South India Shelf Province (Fig. 1). The Gulf of Oman and Persian Gulf are two separate ecoregions located in the Arabian Province. Based on rocky intertidal barnacles, the Gulf of Oman Ecoregion should include Gujarat, while the boundary to the Western Indian Ecoregion appears to be adjacent to waters around Mumbai. Similar patterns may emerge from other groups of marine species. Extensive studies on the biogeography of different groups of organisms across these two ecoregions should be conducted.

There are nine species with a very wide geographical distribution in the Indo-Pacific, all of which are recorded in Gujarat. *Lepas anatifera* and *L. anserifera* are pelagic species that attach to floating objects and get carried by ocean currents (Schiffer and Herbig 2016). *Chelonibia testudinaria* is epibiotic on turtle and decapod hosts. Population genetics studies revealed that there are genetic differences among Western Pacific, Eastern Pacific and Western Atlantic populations of *C. testudinaria* (Rawson et al. 2003).

*Amphibalanus amphitrite*, *A. reticulatus*, and *Megabalanus tintinnabulum* are common fouling species that disperse via ballast water or shipping industries. Chen et al. (2014) examined the world-wide genetic differentiation of *A. amphitrite* and identified three molecular clades, which include a worldwide clade (present in most of the world’s oceans); a second clade common in tropical regions; and a third clade that is only found in the Eastern Atlantic waters. The genetic differentiation among fouling barnacles could be a result of the combined effects of historical events such as Pleistocene sea level changes and human-mediated dispersals (Chen et al. 2014).

Some Indo-Pacific species were recorded in the present study. The intertidal barnacle *T. karandei* was first identified in Mumbai, India (Ross 1971); Ross (1972) subsequently recorded it in Taiwan. The present study is the third report of this species in northwest India. *Striatobalanus tenuis* is a widely reported epibiotic species that often attaches to deep-water crustaceans and mollusc shells. *Microeuraphia withersi* is a high shore chthamalid barnacle common on shaded regions of the Indo-Pacific rocky shores (Poltarukha 1997). There are currently no genetic studies on the diversity or population genetics of these species. It is possible that cryptic species are present among these nominal species across the Indo-Pacific region, like for many other barnacles, as well as for hermit crabs and other decapods (Chan et al. 2007; Tsang et al. 2012; Jung et al. 2018; Shih and Poupin 2020) or that they are homogeneous populations across large geographical expanses (see example of intertidal blennies in Hongjamrassilp et al. 2020). Future research should also focus on the diversity and biogeography of rhizocephalan species in India, as this superorder of barnacles remains extremely understudied in India. It is possible that Indian rhizocephalan species are present in decapods and hermit crabs and exhibit distinct biogeographical distributions similar to the patterns recognised in the Northwest Pacific (Jung et al. 2019).

## Supplementary Material

XML Treatment for Striatobalanus tenuis

XML Treatment for Amphibalanus amphitrite

XML Treatment for Amphibalanus reticulatus

XML Treatment for Megabalanus tintinnabulum

XML Treatment for Chelonibia testudinaria

XML Treatment for Tetraclita ehsani

XML Treatment for Tetraclitella karandei

XML Treatment for Chthamalus barnesi

XML Treatment for Microeuraphia withersi

XML Treatment for Lepas anatifera

XML Treatment for Lepas anserifera
